# Microbial dynamics during harmful dinoflagellate *Ostreopsis* cf. *ovata* growth: Bacterial succession and viral abundance pattern

**DOI:** 10.1002/mbo3.584

**Published:** 2018-02-27

**Authors:** Flavio Guidi, Laura Pezzolesi, Silvana Vanucci

**Affiliations:** ^1^ Department of Biological, Geological and Environmental Sciences (BiGeA) University of Bologna Ravenna Italy; ^2^ Department of Chemical, Biological, Pharmaceutical and Environmental Sciences (ChiBioFarAm) University of Messina Messina Italy

**Keywords:** algal–bacterial associations, *Dinoroseobacter*, harmful dinoflagellates, *Oceanicaulis*, *Ostreopsis*, viruses

## Abstract

Algal–bacterial interactions play a major role in shaping diversity of algal associated bacterial communities. Temporal variation in bacterial phylogenetic composition reflects changes of these complex interactions which occur during the algal growth cycle as well as throughout the lifetime of algal blooms. Viruses are also known to cause shifts in bacterial community diversity which could affect algal bloom phases. This study investigated on changes of bacterial and viral abundances, bacterial physiological status, and on bacterial successional pattern associated with the harmful benthic dinoflagellate *Ostreopsis* cf. *ovata* in batch cultures over the algal growth cycle. Bacterial community phylogenetic structure was assessed by 16S rRNA gene ION torrent sequencing. A comparison between bacterial community retrieved in cultures and that one co‐occurring in situ during the development of the *O*. cf. *ovata* bloom from where the algal strain was isolated was also reported. Bacterial community growth was characterized by a biphasic pattern with the highest contributions (~60%) of highly active bacteria found at the two bacterial exponential growth steps. An alphaproteobacterial consortium composed by the Rhodobacteraceae *Dinoroseobacter* (22.2%–35.4%) and *Roseovarius* (5.7%–18.3%), together with *Oceanicaulis* (14.2‐40.3%), was strongly associated with *O*. cf. *ovata* over the algal growth. The Rhodobacteraceae members encompassed phylotypes with an assessed mutualistic‐pathogenic bimodal behavior. *Fabibacter* (0.7%–25.2%), *Labrenzia* (5.6%–24.3%), and *Dietzia* (0.04%–1.7%) were relevant at the stationary phase. Overall, the successional pattern and the metabolic and functional traits of the bacterial community retrieved in culture mirror those ones underpinning *O*. cf. *ovata* bloom dynamics in field. Viral abundances increased synoptically with bacterial abundances during the first bacterial exponential growth step while being stationary during the second step. Microbial trends also suggest that viruses induced some shifts in bacterial community composition.

## 1 INTRODUCTION

Over the last few decades, a worldwide increase in the occurrence, geographic expansion, and persistence of harmful algal blooms (HABs) has been reported (Anderson, Cembella, & Hallegraeff, 2012; Berdalet et al., 2017; Hallegraeff, 1993; Sunda, Graneli, & Gobler, 2006) with consequent severe impacts on human health and coastal ecosystem services (i.e., fisheries, tourism, and recreation) (Berdalet et al., 2016; Davidson, Tett, & Gowen, 2011; Van Dolah, 2000). While most research has focused on physical and chemical factors forcing HABs dynamics (e.g., Accoroni & Totti, 2016; Davidson et al., 2014; Figueiras, Pitcher, & Estrada, 2006; Van Dolah, 2000), an increasing interest on the interactions between microalgae and bacteria in regulating HABs (Doucette, Kodama, Franca, & Gallacher, 1998; Jones, Mikulski, Barnhorst, & Doucette, 2010; Kodama, Doucette, & Green, 2006; Meyer, O'Neil, Hitchcock, & Heil, 2014; Vanucci, Guidi, Pistocchi, & Long, 2016; Yang et al., 2015) and toxins production (Kodama et al., 2006 and references therein; Green, Hart, Blackburn, & Bolch, 2010; Albinsson, Negri, Blackburn, & Bolch, 2014; Sison‐Mangus, Jiang, Tran, & Kudela, 2014) has developed.

In natural aquatic environments, microalgae and bacteria grow in close association engaging complex interactions (reviewed in Ramanan, Kim, Cho, Oh, & Kim, 2016). Algal–bacterial interactions change during the algal growth cycle (Bolch, Bejoy, & Green, 2017; Mayali & Doucette, 2002; Mayali, Franks, & Azam, 2007; Wang, Tomasch, Jarek, & Wagner‐Döbler, 2014) as well as throughout the lifetime of the blooms, including the harmful ones (Buchan, Lecleir, Gulvik, & González, 2014; Fandino, Riemann, Steward, Long, & Azam, 2001; Mayali, Franks, & Burton, 2011), affecting the dynamics of these events. In parallel, changes of these interactions play a major role in shaping diversity and structure of algal‐associated bacterial communities (Bagatini et al., 2014; Grossart, Levold, Allgaier, Simon, & Brinkhoff, 2005; Teeling et al., 2012). Actually, microalgae and bacteria reciprocally affect their physiology and metabolism (Albinsson et al., 2014; Bolch, Subramanian, & Green, 2011; Bolch et al., 2017; Jauzein, Evans, & Erdner, 2015) through relationships which range from mutualistic to antagonistic (Amin, Parker, & Armbrust, 2012; Cooper & Smith, 2015; Ramanan et al., 2016). A mutualistic interaction relying upon the exchange of beneficial compounds has been proposed for members of Alphaproteobacteria in relation to different algal bloom‐forming species. Specifically, the bacteria provide essential molecules (e.g., B vitamins and growth promoting factors) and antibiotics effective against algal pathogens in return for algal fixed carbon exudates (primarily dimethylsulfoniopropionate and Krebs cycle intermediates; Wagner‐Döbler et al., 2010; Seyedsayamdost, Case, Kolter, & Clardy, 2011; Wang et al., 2014, 2015; Amin et al., 2015; Cruz‐López & Maske, 2016; Segev et al., 2016; Wang, Gallant, & Seyedsayamdost, 2016). Furthermore, bacterial phylotypes belonging to Rhodobacteraceae have been found to switch from mutualists to pathogens of their dinoflagellate hosts in response to either photosynthetic products or algal senescence signaling molecules (Riclea et al., 2012; Segev et al., 2016; Seyedsayamdost et al., 2011; Sule & Belas, 2013; Wang et al., 2014, 2015, 2016). These findings imply a possible relevance of this bimodal interaction in algal bloom initiation and termination (Riclea et al., 2012; Wang et al., 2014), especially once the dominance of the same phylotypes is assessed in the bacterial communities associated with both bloom phases. In addition, quality and amount of the algal‐released compounds, that depend on algal species and its physiological status, would define phylogenetic structure (Bennke, Neu, Fuchs, & Amann, 2013; Christie‐Oleza, Scanlan, & Armengaud, 2015; Xing et al., 2015) and successional pattern of the associated bacterial community (e.g., Bagatini et al., 2014; Grossart et al., 2005; Teeling et al., 2012).

A deep knowledge on phylogenetic composition and successional dynamics of bacterial communities associated with HABs is therefore recognized as a crucial step for unveiling relevant and recurrent algal‐bacterial associations underpinning the different bloom phases (Bagatini et al., 2014; Mayali et al., 2011; Tada, Taniguchi, Sato‐Takabe, & Hamasaki, 2012; Yang et al., 2015), and in parallel with complementing laboratory‐based studies, it will allow to elucidate the functional significance of these complex interactions (Bagatini et al., 2014; Buchan et al., 2014; Kazamia, Helliwell, Purton, & Smith, 2016; Sison‐Mangus et al., 2014). Indeed, although 16S rRNA gene phylogenetic surveys do not directly decode bacterial functionality, they still provide insights on how the different bacterial groups correlate within the assemblages and with the microalgal partner, considering certain metabolic characteristics significant to the groups and to the associated organism (Amin et al., 2012; Buchan et al., 2014; Gifford, Sharma, & Moran, 2014; Newton et al., 2010). Next Generation Sequencing approaches typically allow a deeper phylogenetic analysis than traditional molecular methods, used in most of the available studies describing bacterial communities associated with toxic dinoflagellates (Garcés et al., 2007; Jones et al., 2010; Mayali et al., 2011; Park et al., 2015; Yang, Zhou, Zheng, Tian, & Zheng, 2012), therefore considerably reducing the gap of knowledge on this topic.

In the last decade, the increasing occurrence of extensive *Ostreopsis* cf. *ovata* Fukuyo blooms has been reported in temperate coastal regions, including the Mediterranean (Accoroni & Totti, 2016; Aligizaki & Nikolaidis, 2006; Funari, Manganelli, & Testai, 2015; Mangialajo et al., 2011; Vila, Garcés, & Masó, 2001). Mediterranean *O*. cf. *ovata* produces palytoxin‐like compounds, namely, isobaric palytoxin and a wide range of ovatoxins (OVTX‐a to ‐k; Ciminiello et al., 2012; Brissard et al., 2015; Tartaglione et al., 2016) under both field (Accoroni et al., 2011; Carnicer, Guallar, Andree, Diogène, & Fernández‐Tejedor, 2015; Ciminiello et al., 2006, 2008) and laboratory conditions (Pezzolesi et al., 2014, 2016; Pistocchi et al., 2011; Vanucci, Pezzolesi, et al., 2012; Vanucci, Guerrini, et al., 2012). The epiphytic/benthic dinoflagellate grows onto a wide range of substrata, forming thick mucilaginous mats (Giussani et al., 2015; Honsell et al., 2013; Totti, Accoroni, Cerino, Cucchiari, & Romagnoli, 2010). Blooms occur during summer‐late summer often in moderate anthropogenic impacted sites (Accoroni & Totti, 2016; Accoroni et al., 2015; Marini, Fornasiero, & Artegiani, 2002), and they can have a severe impact on human health (Funari et al., 2015; Kermarec et al., 2008 and references therein) and on invertebrate benthic communities (Accoroni et al., 2011; Carella et al., 2015; Migliaccio et al., 2016). Toxin accumulation has been reported in macrofauna (Aligizaki, Katikou, Milandri, & Diogene, 2011; Biré et al., 2015; Furlan et al., 2013), yet no connected food poisoning has been shown.

Recently, pyrosequencing analysis revealed that Rhodobacteraceae members belonging to the genera *Ruegeria*,* Jannaschia*, and *Roseovarius* were dominant at both development and maintenance/decline phases of an *O*. cf. *ovata* natural bloom, suggesting a bimodal behavior of these phylotypes. Whereas, secondary colonizer bacteria such as Flavobacteria‐Sphingobacteria and Actinobacteria increased in abundance at the maintenance/decline phase of the bloom (Vanucci, Guidi, et al., 2016). During the aforementioned bloom, however, epiphytic diatoms co‐occurred in relevant proportion in the *O*. cf. *ovata* mats, as found in other *O*. cf. *ovata* blooms (Accoroni, Romagnoli, Pichierri, & Totti, 2016; Totti et al., 2010) also from different geographic areas (Aligizaki & Nikolaidis, 2006; Carnicer et al., 2015; Vila et al., 2001). Thus, the isolation of *O*. cf. *ovata* cells from the natural bloom and the setting up of culture‐based studies appear fundamental steps in the attempt to discern the more intimate and recurrent bacteria interacting with the dinoflagellate.

This study assessed the bacterial diversity associated with *O*. cf. *ovata* in batch cultures with the aim of elucidating the most prominent microbial associations. Temporal changes in bacterial abundance together with phylogenetic successional pattern were followed over the different algal growth phases, highlighting shifts in bacterial community composition with possible ecological and functional significance on *O*. cf. *ovata* growth dynamics. Moreover, a comparison between *O*. cf. *ovata* associated bacterial community over the different algal growth phases and that one co‐occurring in situ during the evolvement of the *O*. cf. *ovata* bloom from where the algal strain was isolated was also reported. Level of phylogenetic overlapping and functional redundancy between the two communities was assayed, in order to evaluate the reliability of laboratory cultures for future manipulative experiments.

Bacterial phylogenetic composition was recovered by high‐throughput parallel tag sequencing using ION torrent PGM platform. Additionally, the highly respiring bacteria were identified as those ones able to reduce the fluorogenic redox dye 5‐cyano‐2,3‐dytolyl tetrazolium chloride (CTC), in order to provide details on bacterial community's physiological status during cultures progression.

The presence of viruses and their abundance pattern were also evaluated synoptically throughout the *O*. cf. *ovata* growth. Actually, it is known that bacterial communities are also shaped in terms of diversity and dynamics by viral activity, mainly affecting the most abundant and metabolically active species (Del Giorgio & Gasol, 2008; Fuhrman, 1999; Sime‐Ngando, 2014; Wommack & Colwell, 2000 and references therein). However, viruses have been seldom taken into consideration in HABs dynamics (Loureiro, Reñé, Garcés, Camp, & Vaqué, 2011; Meyer et al., 2014).

To the best of our knowledge, this is the first study that provides viral and highly respiring bacterial cells (CTC^+^ cells) abundance trends, as well as bacterial 16S rRNA gene Next Generation Sequencing data associated with a cultured toxic dinoflagellate.

## 2 MATERIALS AND METHODS

### 2.1 Experimental setup and culture conditions

*O*. cf. *ovata* strain OOAP1209 was isolated in September 2012 from macrophyte samples collected at the early phase of an *O*. cf. *ovata* bloom along the coast of North‐western Adriatic Sea (Passetto, Italy, 43°36′38″ N and 13°32′20″ E; Vanucci, Guidi, et al., 2016), using capillary pipette method under sterile conditions (Hoshaw & Rosowski, 1973), and using 0.22‐μm‐pore‐size filtered and autoclaved seawater for cell washing steps. After initial growth in microplates, cells were maintained in sterile flasks sealed with cotton plugs at 20°C ± 1°C under a 16:8 hr light:dark cycle in a growth chamber (photon flux density 110–120 μmol m^2^ s^−1^ by cool white lamp).

Cultures were set up in sterile f/2 medium (minus silicate) (Guillard, 1975) plus selenium, with macronutrients (NO_3_ˉ and PO_4_^3^ˉ) added at a fivefold diluted concentration. The medium was prepared from natural seawater kept several weeks in the dark before use. The seawater was 0.22‐μm‐pore‐size filtered and autoclaved, and adjusted to salinity value of 36. Medium also contains trace metals and vitamins (Guillard, 1975).

Experimental cultures consisted in triplicate 3‐L Erlenmeyer flasks, inoculated with *O*. cf. *ovata* collected from a culture at end exponential/early stationary phase and fresh medium to a final volume of 2650 mL, in order to have a concentration of about 300 cells mL^−1^ at the beginning of the experiment (day 0). All experimental manipulations were carried out under a laminar flow hood using sterile equipment.

Aliquots for *O*. cf. *ovata* enumeration, bacterial and viral enumeration, and for assessment of bacterial physiological status were collected at day 0, 3, 6, 9, 12, 18, 24, 32, and 42. Aliquots for nutrient analysis were collected at day 0, 3, 6, 9, 12, 24, and 42, whereas aliquots for phylogenetic analysis of the bacterial community were collected at day 0, 6, 24, and 42. For all the analyses, aliquots were collected from each flask in triplicate.

### 2.2 *Ostreopsis* cf. *ovata* enumeration and nutrient analysis

*O*. cf. *ovata* cell counts were carried out following Utermöhl method (Hasle, 1978) using a Zeiss Axiovert 100 inverted microscope at 320× magnification under bright field and phase contrast illumination. Specific growth rate (μ, day^−1^) was calculated using the following equation:

$$\mu =\frac{\ln N_{1}-\;\ln N_{0}}{t_{1}-\;t_{0}}$$

where *N*_0_ and *N*_1_ were cell density values (cells mL^−1^) at time *t*_0_ and *t*_1_.

Nitrate and phosphate analyses were performed on filtered culture medium aliquots (Whatman GF/F filters, pore size 0.7 μm) and analyzed spectrophotometrically (UV/VIS, JASCO 7800, Tokyo, Japan) according to Strickland and Parsons (1972).

### 2.3 Bacterial and viral enumeration and assessment of bacterial physiological status

Bacterial and virus like particles (VLPs) abundances were determined in the same culture aliquots fixed with 0.02 μm prefiltered formaldehyde (2%), following the method described by Shibata et al. (2006). Briefly, aliquots (1 mL) were filtered onto 0.02 μm pore size Anodisc filters (Whatman, 25 mm diameter) and stained with 100 μL SYBR Gold (Life Technologies) at 8× final concentration, then mounted onto microscopic slides and stored at −20°C. Bacterial and viral enumerations were performed using epifluorescence microscopy (Nikon Eclipse 80i, magnification 1000×) under blue light excitation, counting at least 20 fields and a minimum of 300 cells per replicate. Viruses were discriminated from bacteria on the basis of their dimensions (Noble & Fuhrman, 1998).

Bacterial physiological status was assessed by determining highly respiring bacteria as those able to reduce 5‐cyano‐2,3‐ditolyl tetrazolium chloride (CTC; Sigma‐Aldrich), which turns into a red fluorescent formazan detectable by epifluorescence microscopy (Sherr, del Giorgio, & Sherr, 1999). Sample aliquots (0.9 mL) were amended with 100 μL of a 50 mmol L^−1^ CTC solution (final concentration 5 mmol L^−1^) immediately following collection and were incubated for 3 hr in the dark at room temperature. After the incubation, samples were fixed with 0.22 μm prefiltered formaldehyde (2%) and then filtered onto 0.22 μm pore size black‐stained polycarbonate membrane filters (Millipore). Cell counts were performed using epifluorescence microscopy as described above for bacteria and VLPs.

### 2.4 Bacterial DNA extraction and PCR amplification

*O*. cf. *ovata* cultures in aliquots of 30–100 mL volume were harvested at the time of inoculum and at exponential, mid and late stationary algal growth phases (day 0, 6, 24, 42, respectively) by filtration under low vacuum onto Supor 200 PES filters (Pall Corporation/Pall Life Sciences, pore size 0.2 μm). All filters were stored at −80°C in sterile 2‐mL centrifuge tubes until analysis. For DNA extraction, filters were shredded under sterile conditions, and DNA from cells on the filters was extracted using the ZR Soil Microbe DNA MiniPrep Kit (Zymo Research) according to the manufacturer's instructions. The extracted DNA samples from the aliquots harvested at the time of inoculum and at each algal growth phase were, respectively, pooled together at equimolar amounts based on their DNA concentrations, thus increasing, at the same time, sample size and the successive depth of sequencing per sample. This procedure was chosen in the attempt to maximize bacterial diversity retrieval and to assess the proper phylotypes’ contribution to the community, avoiding potential biases due to algal mucilage aggregates. *O*. cf. *ovata* cells, in fact, form mucilaginous aggregates increasing in size and abundance during culture progression (Pezzolesi et al., 2014; Vanucci, Pezzolesi, et al., 2012) and being unevenly distributed among the different aliquots. It is known that the mucilage layer harbors some selective fractions of the dinoflagellate bacterial community co‐occurring with *O*. cf. *ovata* bloom (Vanucci, Guidi, et al., 2016).

Partial bacterial 16S rRNA genes (hypervariable V1‐V2 region) were amplified using universal bacterial primers 8F (5′‐AGAGTTTGATCCTGGCTCAG‐3′) and 338R (5′‐GCTGCCTCCCGTAGGAGT‐3′) and master mixes prepared with Qiagen Hotstar Hi‐Fidelity Polymerase Kit (Qiagen). Amplification in triplicate of each sample was performed with following conditions: an initial denaturing step at 94°C for 5 min, followed by 27 cycles of denaturing of 94°C for 45 s, annealing at 50°C for 30 s and a 1 min 30 s extension at 72°C, ending with a 10 min extension at 72°C and a final hold at 4°C. Each amplification was checked by electrophoresis on a 2% agarose gel. In order to remove primer dimers, the replicate PCR reactions were pooled and purified using Agencourt AMPure XP PCR purification kit (Beckman Coulter Inc.) according to the manufacturer's instructions. Purified amplicons were quantified using a Bioanalyzer High Sensitivity DNA Kit (Agilent Technologies) and pooled at equimolar ratio. Barcoded amplicon libraries were realized using the Ion Plus Library Kit and Ion Xpress Barcode Adapters (Life Technologies) in preparation for clonal amplification. Emulsion PCR was performed using the Ion PGM Template OT2 400 Kit (Life Technologies) according to the manufacturer's instructions. Multiplexed sequencing of the amplicon libraries was carried out on a 316 v2 chip with the Ion Torrent PGM system using 850 flows and employing the Ion PGM Sequencing 400 Kit (Life Technologies) according to the supplier's instructions.

After sequencing, the individual sequence reads were filtered by the PGM software for low quality and polyclonal sequences removal. Sequences matching the PGM 3′ adaptor were also automatically trimmed. All PGM quality approved, trimmed and filtered data were exported as fastq files.

### 2.5 Sequence processing and diversity analysis

The fastq files were processed using MOTHUR (Schloss et al., 2009). Quality control retained sequences with a length between 250 and 400 bp, average sequence quality score >25, with truncation of a sequence at the first base if a low‐quality rolling 10 bp window was found. Sequences with presence of homopolymers >6 bp, any ambiguous base call, mismatched primers and more than one error on barcode sequence were omitted. Community taxonomy information was obtained using a Ribosomal Database Project naive Bayesian rRNA classifier (Wang, Garrity, Tiedje, & Cole, 2007), and those sequences either related to chloroplasts and mitochondria or not belonging to the Domain Bacteria were discarded from the dataset. The remained unique sequences were aligned against the Silva bacteria database. After screening, filtering, preclustering, and chimera removal, samples were standardized to the size of the smallest library (15,270 reads) by randomly subsampling datasets, and the retained sequences were used to build a distance matrix. Bacterial sequences were grouped into operational taxonomic units (OTUs) by clustering at 97% similarity, then singleton OTUs were discarded from the analysis if they were not found in at least two different samples. The representative sequences for each OTU were picked and classified using the RDP classifier. OTUs were defined as *abundant* when representing ≥1% of the community in at least one of the samples, and as *rare* when their relative abundance was <1%. Bacterial community diversity was addressed through three diversity indices (observed OTU richness, Shannon diversity and Good's coverage) and a Bray–Curtis similarity matrix of OTU abundance data was performed in PAST 3.14 (Hammer, Harper, & Ryan, 2001).

Sequences were submitted to GenBank with the project reference (BioProject ID) PRJNA339161.

### 2.6 Statistical analysis

All statistical analyses were performed with PAST 3.14. Differences in the investigated variables were tested by the analysis of variance (ANOVA). Statistical significance was set at *p *<* *.05 for all the analyses.

## 3 RESULTS

### 3.1 *Ostreopsis* cf. *ovata* cell growth, bacterial abundance and physiological status, viral abundance

**Figure 1:**
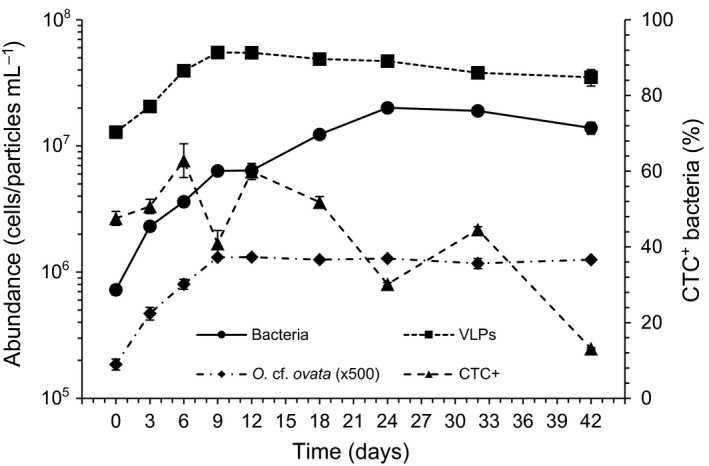
Growth pattern of *Ostreopsis* cf. *ovata* and bacterial cells, temporal trend of the contribution (%) of highly respiring bacterial cells (CTC ^+^ cells) to the total bacterial abundance, and viral abundance (VLPs) trend. *O*. cf. *ovata* abundances are multiplied by a factor of 500. Each point is the mean of triplicate cultures. Bars indicate standard deviations

Growth curve of *O*. cf. *ovata* is shown in Figure 1. Cultures initial cell densities were 372 ± 37 cells mL^−1^; the exponential phase ended by day 9 (mean growth rate: 0.22 ± 0.01 day^−1^) attaining to a cell yield of 2.63 × 10^3^ ± 9.55 × 10^1^ cells mL^−1^, and at the stationary phase an increase in mucilaginous cell aggregates was evident.

Inorganic nutrients (i.e., NO_3_ˉ and PO_4_^3^ˉ) were rapidly taken up by the cells during the first days of growth, and being almost depleted by day 12 (Figure S1).

Over the *O*. cf. *ovata* growth cycle bacterial cell densities increased by more than one order of magnitude (range: 7.24 × 10^5^ to 2.01 × 10^7^ cells mL^−1^, day 0 and 24, respectively; mean value: 9.41 × 10^6^ ± 6.77 × 10^6^ cells mL^−1^). Bacterial community growth was characterized by a biphasic pattern (Figure 1): a first exponential phase (first bacterial growth step) occurred synoptically with the algal exponential growth phase (i.e., days 0–9), whereas a second exponential phase (second bacterial growth step) occurred between day 12 and day 24 of the algal mid stationary phase (days 9–24), and it was characterized by a lower bacterial growth rate with respect to the first one (μ = 0.24 and 0.10 day^−1^, days 0–9 and 12–24, respectively; ANOVA, *p *<* *.01).

Contribution of highly respiring bacterial cells (CTC^+^ cells) to the total bacterial abundance was, on average, 45 ± 15% (Figure 1). Particularly, the highest CTC^+^ cells relative abundances were found synoptically with the first and the beginning of the second bacterial exponential steps (62.8% and 59.9%, day 6 and 12, respectively), whereas a significant drop in CTC^+^ cells contribution was recorded at day 9 (Figure 1; ANOVA, *p *<* *.05). A global decreasing trend in CTC^+^ cells contribution was then observed after day 12, with another drop concomitant with the end of the second bacterial exponential step (day 24; ANOVA, *p *<* *.01), and reaching values of about 13% at the end of the experiment (day 42).

Abundance of virus like particles (VLPs) ranged between 1.29 × 10^7^ and 5.50 × 10^7^ VLPs mL^−1^ (day 0 and 9, respectively), showing a fourfold higher mean value (3.86 × 10^7^ ± 1.33 × 10^7^ VLPs mL^−1^) than bacterial one. While during the first bacterial growth step (days 0–9) viral abundances exhibited a synoptic increasing pattern, during the second bacterial growth step and afterward they were almost stationary, slightly decreasing (Figure 1). The consequent mean virus to bacteria ratios (VBR, Figure S2) were equal to 11.4 between days 0–9, to 4.9 between days 12–24 and to 2.3 between days 32–42.

### 3.2 Bacterial community diversity and phylogenetic composition

**Figure 2:**
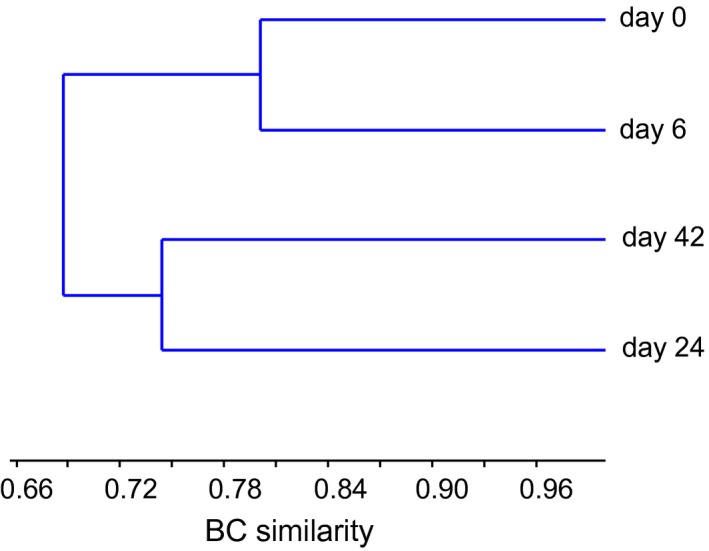
UPGMA cluster of bacterial community's structure using Bray–Curtis (BC) distances. Samples were collected at the time of inoculum (day 0) and during the different algal growth phases (days 6, 24, and 42). The dendrogram was constructed on the basis of bacterial OTU abundances retrieved from ION torrent sequencing data. Total similarity between samples is indicated by 1, and total dissimilarity is indicated by 0

Overall, 247,426 high‐quality reads spanning the hypervariable regions V1–V2 of the bacterial 16S rRNA gene were obtained (average length = 290 bp), yielding 207 OTUs after normalization on the smallest sample size (15,270 reads), singletons removal, and chloroplast and mitochondrial sequences discharge. Rarefaction curves (Figure S3) as well as coverage values (Table 1) revealed that most of the bacterial diversity was recovered by sequencing analysis. The unweighted pair group method with arithmetic mean (UPGMA) dendrogram of Bray–Curtis distances between samples showed that samples collected at earlier (i.e., day 0 and 6) and later (i.e., day 24 and 42) algal growth phases formed two distinct clusters although differences were not significant (Figure 2; ANOSIM, *p *>* *.05), nevertheless suggesting a shift in bacterial OTU composition during algal growth proceeding.

**Table 1:** Bacterial diversity parameters during the *Ostreopsis* cf. *ovata* growth. Summary of total sequences after normalization (Reads), richness as number of bacterial operational taxonomic units detected at 97% identity (OTUs), Shannon diversity (H’), and Good's sample coverage obtained by Ion torrent sequencing data at the time of inoculum (day 0) and during the different algal growth phases (days 6, 24, and 42)

Sample	Reads	OTUs	H′	Good's coverage (%)
Day 0	15,270	202	3.17	99.96
Day 6	15,270	195	2.90	99.99
Day 24	15,270	203	3.05	99.79
Day 42	15,270	205	3.12	99.11

**Figure 3:**
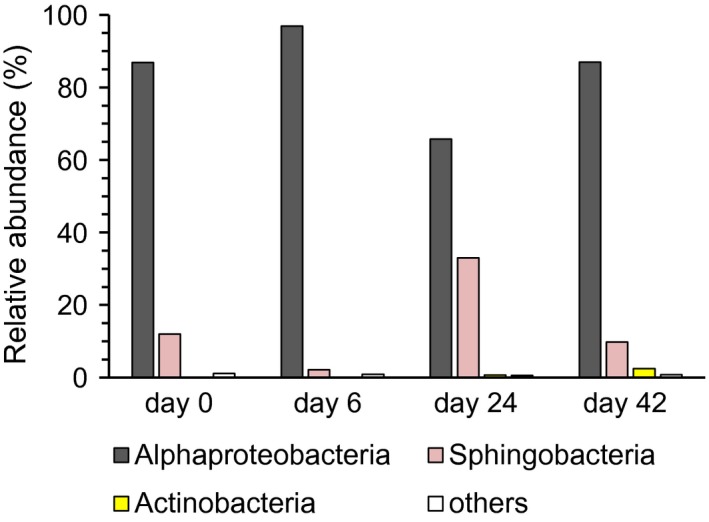
Percent distribution of the dominant classes (≥1% in at least one of the samples) detected in *O*. cf. *ovata* batch cultures at the time of inoculum (day 0) and during the different algal growth phases (days 6, 24, and 42), as revealed from ION torrent sequencing data. “others” represent the classes with less than 1% of relative abundance individually

**Figure 4:**
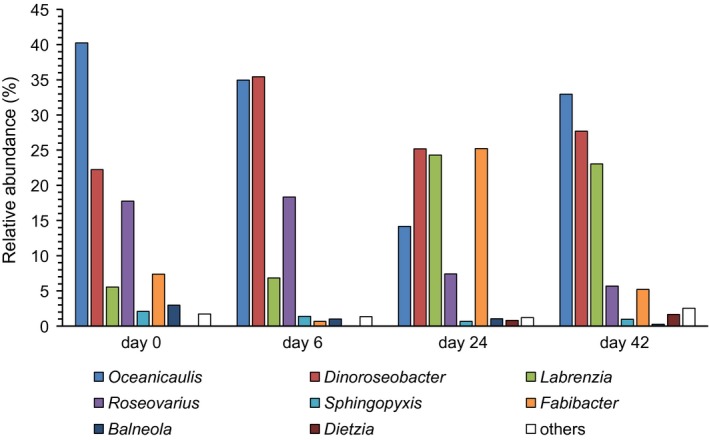
Relative contribution of the major bacterial genera (≥1% in at least one of the samples) retrieved in *O*. cf. *ovata* batch cultures at the time of inoculum (day 0) and during the different algal growth phases (days 6, 24, and 42), as revealed from ION torrent sequencing data. “others” represent the genera with less than 1% of relative abundance individually

Bacterial diversity retrieved by ION torrent analysis over the *O*. cf. *ovata* growth cycle was spread across 5 phyla, 7 classes and 21 genera (Tables S1–S3). Alphaproteobacteria (range: 65.7%–96.9%, day 24 and 6, respectively), Sphingobacteria (2.2%–33.0%, day 6 and 24) and Actinobacteria (0.04%–2.5%, day 0 and 42) dominated the community, together accounting for more than 98% in relative contribution in all samples (Figure 3). Alphaproteobacteria was the most abundant class throughout the algal growth (ANOVA, *p *<* *.05), being mostly represented by the genus *Oceanicaulis* (fam. Hyphomonadaceae; range: 14.2%–40.3%, day 24 and 0, respectively) and the Rhodobacteraceae affiliated genera *Dinoroseobacter* (22.2%–35.4%, day 0 and 6), *Roseovarius* (5.7%–18.3%, day 42 and 6), and *Labrenzia* (5.6%–24.3%, day 0 and 24). Sphingobacteria and Actinobacteria were mainly represented by members of the genera *Fabibacter* (fam. Flammeovirgaceae; 0.7%–25.2%, day 6 and 24) and *Dietzia* (0.04–1.7%, day 0 and 42), respectively (Figure 4). Changes of the main bacterial taxa during *O*. cf. *ovata* growth mostly relied upon the contribution of the 14 abundant OTUs (i.e., ≥1% of the total reads in at least one of the samples; Tables 2 and 3), together accounting for 78%–82% of the total community in all samples. While at the time of inoculum (day 0) OTUs #2, #9, #13, #18, and #20 affiliated to *Oceanicaulis* showed the highest contribution (18.5% for OTU #2, 2.1%–5.3% for the others), during the algal exponential growth phase (i.e., first bacterial growth step) the overall most abundant *Dinoroseobacter‐*related OTU #1 represented almost a third of the total community (29.2%), showing the highest percentage together with *Roseovarius* OTUs #7, #11 (11.2% and 5.9%, respectively) and OTU #9 (7.7%). At the *O*. cf. *ovata* mid stationary phase (i.e., end of the second bacterial growth step, day 24), *Fabibacter* and *Labrenzia*‐related OTUs #6 and #4 (21.9% and 16.9%, respectively) together with OTU #1 (19.0%) made up more than half of the total bacteria. Lastly, at the *O*. cf. *ovata* late stationary phase (day 42) *Dinoroseobacter* and *Oceanicaulis* restored the dominance observed at the exponential phase (day 6), whereas OTU #4 still contributed for ~17% to the total community (Table 2). Gammaproteobacteria were rare (<1%) at all *O*. cf. *ovata* growth phases, with *Alcanivorax* being the main representative of this class (0.6%, day 6; Table S2 and S3).

**Table 2:** Relative contribution (%) of the abundant OTUs (≥1% of the total reads in at least one of the samples) retrieved at the time of inoculum (day 0) and during the different algal growth phases (days 6, 24, and 42), as revealed by ION torrent sequencing data

OTU #	Closest relative (RDP classifier)	Day 0	Day 6	Day 24	Day 42
1	*Dinoroseobacter*	18.1	29.2	19.0	22.5
2	*Oceanicaulis*	18.5	14.3	4.4	13.9
4	*Labrenzia*	4.3	5.1	16.9	16.7
6	*Fabibacter*	6.8	0.6	21.9	4.8
7	*Roseovarius*	11.0	11.2	4.1	3.6
9	*Oceanicaulis*	5.3	7.7	3.4	6.5
11	*Roseovarius*	5.3	5.9	2.3	1.7
13	*Oceanicaulis*	3.1	2.2	0.9	2.2
16	*Dietzia*	0.0	0.0	0.7	1.5
18	*Oceanicaulis*	2.3	2.1	0.8	1.8
20	*Oceanicaulis*	2.1	1.4	0.5	1.4
21	Flammeovirgaceae	0.1	0.0	1.6	1.5
33	Rhodobacteraceae	1.3	1.5	0.9	0.3
35	*Balneola*	2.0	0.7	0.7	0.2

**Table 3:** Closest matches from the NCBI GenBank database based on sequence similarity of the most abundant OTUs (≥1% of the total reads in at least one of the samples) as revealed by Ion torrent sequencing data

OTU#	Closest matched sequence (% 16S rRNA gene similarity)	NCBI accession number	Closest cultured neighbor (% 16S rRNA gene similarity)	NCBI accession number
1	*Dinoroseobacter shibae* (99)	NR074166.1	*Dinoroseobacter shibae* (99)	NR074166.1
2, 9, 13, 18, 20	Uncultured bacterium (99)	JQ337901.1	*Oceanicaulis alexandrii* (96)	NR025456.1
4	*Labrenzia alexandrii* (100)	NR042201.1	*Labrenzia alexandrii* (100)	NR042201.1
6	Uncultured bacterium (99)	JX016873.1	*Fabibacter pacificus* (92)	NR109732.1
7, 11	*Roseovarius* sp. (100)	AB114422.1	*Roseovarius tolerans* (99)	NR026405.1
16	Uncultured bacterium (98)	FJ594833.1	*Dietzia cinnamea* (81)	NR116686.1
21	*Reichenbachiella* sp. (94)	JX854345.1	*Reichenbachiella faecimaris* (93)	NR117445.1
33	*Thalassococcus lentus* (100)	NR109663.1	*Thalassococcus lentus* (100)	NR109663.1
35	Uncultured *Balneola* sp. (98)	JX529426.1	*Balneola alkaliphila* (95)	NR044367.1

## 4 DISCUSSION

### 4.1 Microbial dynamics during *Ostreopsis* cf. *ovata* growth

Growth pattern of *O*. cf. *ovata* and nutrients temporal trend observed in this study are consistent with those previously described for the same algal species under comparable culture conditions (Pezzolesi et al., 2014, 2016; Vanucci, Pezzolesi, et al., 2012). Bacterial community growth showed a biphasic pattern, characterized by two exponential growth steps having different growth rates that appear mainly triggered by different quality and amount of available substrate. The first and faster growth step, occurring synoptically with the algal exponential growth phase, suggests a rapid utilization of the available inorganic nutrients present in the medium not only by *O*. cf. *ovata*, but also by bacteria along with photosynthetic products, mostly of low molecular weight (Buchan et al., 2014; Wagner‐Döbler et al., 2010). Whereas, the second and slower bacterial growth step, occurring at the algal mid stationary phase, suggests the proliferation of bacteria able to grow on a wider pool of algal‐derived organic matter including high molecular weight compounds (Buchan et al., 2014; Thornton, 2014) under low inorganic nutrients concentrations in the culture medium. Accordingly, the highest contributions of highly respiring bacteria (CTC^+^ cells) to the community (~60% of the total bacterial cells) occurred at these two growth steps, whereas the lowest ones (~13%) occurred at late stationary phase, remarking the recognized correlation existing between CTC‐based estimates and bacterial growth rate (e.g., Del Giorgio & Gasol, 2008; Paoli, Karuza, De Vittor, Del Negro, & Fonda Umani, 2006; Sherr et al., 1999). At the same time, the low proportion of CTC^+^ cells found at the end of the experiment does not necessarily indicate high cell mortality. In fact, while CTC^+^ cells represent those bacteria characterized by a high level of metabolic activity, cells showing no apparent CTC reduction can still have different levels of metabolic activity linked to substrate quality (e.g., refractory organic matter) and/or bacterial phylogenetic affiliations (Del Giorgio & Gasol, 2008). So that, changes in bacterial physiological status and/or phylogenetic structure could likely be the reason for the low CTC^+^ cells values at late stationary phase (see also forward). Moreover, as general, the high contributions of active bacteria reported in this study are rarely detectable in natural environments, where high metabolic bacterial cells are selectively grazed (Jürgens & Massana, 2008), besides being preferentially targeted by virus to ensure successful propagation of the progeny (Del Giorgio & Gasol, 2008; Sime‐Ngando, 2014; Wommack & Colwell, 2000). Given this premise, the CTC^+^ cells pattern retrieved here yet resembles bacterial production trend found during a bloom of the toxic planktonic dinoflagellate *Karenia brevis*, where the rates of leucine and thymidine uptake increased and then decreased in line with the initiation and maintenance bloom phases, respectively (Meyer et al., 2014). In addition, temporal microbial (CTC^+^ bacteria and viruses) patterns and the decreasing trend of the mean virus to bacteria ratio (VBR: from 11.4 to 2.3, days 0–9 and 32–42, respectively) indicate a more relevant viral top‐down control (e.g., Meyer et al., 2014; Wommack & Colwell, 2000) at the first bacterial growth step than afterward, suggesting that viruses likely affected the bacterial community composition by impacting most active bacteria rather than affecting the alga straightly, in accordance with previous reports on bloom dynamics of *Karenia brevis* (Meyer et al., 2014; Paul et al., 2002). Nevertheless, further studies are needed to assess a possible presence of algal viruses (and their forms of infection) and its relative importance in the microbial dynamics. Bacterial and viral temporal patterns in this study also suggest a more tight relationship between viral abundance and bacterial growth rate rather than between viral and bacterial abundances, as similarly found in natural environments (e.g., Corinaldesi et al., 2003; Danovaro, Corinaldesi, Filippini, Fischer, & Gessner, 2008; Danovaro et al., 2011; Del Giorgio & Gasol, 2008; Middelboe, 2000; Sime‐Ngando, 2014). Consistently, a higher relative abundance of fast‐growing bacteria was retrieved at the first growth step than at the second one and afterward (i.e., Alphaproteobacteria with respect to Sphingobacteria, Figure 3).

### 4.2 Bacterial diversity and successional pattern

In total, more than 200 OTUs at a 97% similarity level were detected in the bacterial community associated with *O*. cf. *ovata* over the algal growth cycle. As it was expected, ION torrent 16S rRNA gene sequencing revealed a higher bacterial richness than those found for bacterial communities associated with cultured toxic dinoflagellates available to date and assessed with traditional molecular methods (e.g., *Alexandrium* spp., Sala et al., 2005; Pérez‐Guzmán, Perez‐Matos, Rosado, Tosteson, & Govind, 2008; *O. ovata* and *Coolia monotis*, Ruh et al., 2009; *Gymnodinium catenatum*, Green et al., 2010; *Pyrodinium bahamense*, Onda, Azanza, & Lluisma, 2015), while being consistent with the OTU richness values retrieved by next generation sequencing of the bacterial communities associated with single algal cell isolates (Sison‐Mangus et al., 2014). Whereas, OTU richness and Shannon diversity in cultures (range values: 195–205 and 2.90–3.17, respectively; Table 1) were lower than those ones found for the bacterial community co‐occurring with the *O*. cf. *ovata* natural bloom from where the algal strain studied here was isolated (range values: 1621–2214 and 5.28–6.36, OTU richness and Shannon diversity, respectively; Vanucci, Guidi, et al., 2016). This finding remarks that algal cells isolation procedure and laboratory maintenance over successive subcultures can reduce bacterial diversity of the community co‐occurring with the alga in the natural environment. In fact, it is known that culture conditions exert some selective pressure, either suppressing or promoting certain bacterial phylotypes, and likely leading to a dynamic balance over time. Specifically, selective forces over repeated transfers could allow the persistence of those bacteria best adapted to exploitation of algal‐derived products under recurring changes in nutrients and from aerobic to anaerobic conditions, and of those phylotypes with a specific importance for the growth and physiology of the algal cells (e.g., Green, Llewellyn, Negri, Blackburn, & Bolch, 2004; Green et al., 2010; Jasti, Sieracki, Poulton, Giewat, & Rooney‐Varga, 2005; Lupette et al., 2016; Schwenk, Nohynek, & Rischer, 2014) (see also forward). Given this premise, bacterial community composition over the *O*. cf. *ovata* growth cycle was spread across 5 phyla, 7 classes, and 21 genera. Alphaproteobacteria (mainly Rhodobacteraceae), followed by Flavobacteria‐Sphingobacteria and Actinobacteria, dominated the community. This broad bacterial composition feature with the dominance of these three taxa is consistent with most bacterial communities co‐occurring in mesocosms/batch cultures (Bagatini et al., 2014; Grossart et al., 2005; Tada et al., 2012) and in nontoxic and toxic natural blooms of dinoflagellates (Buchan et al., 2014; Garcés et al., 2007; Jones et al., 2010; Mayali et al., 2011). Notably, the relative abundances and successional pattern quite resemble those reported during *O*. cf. *ovata* bloom (Vanucci, Guidi, et al., 2016). Actually, Alphaproteobacteria strongly dominated the exponential and bloom development phases in cultures and in situ, respectively (~90% of the total community for both studies), with a persistent presence of Rhodobacteraceae over the algal growth cycle and bloom phases (~40%–60%). Whereas, secondary colonizer bacteria such as Flavobacteria‐Sphingobacteria (~20%–30%) and Actinobacteria (~2%–3%) were important at stationary growth and bloom maintenance/decline phases, respectively.

Overall, in this study, the co‐dominance of *Oceanicaulis* and *Dinoroseobacter* phylotypes (closely related to *Oceanicaulis alexandrii* and *Dinoroseobacter shibae* at 96% and 99% 16S rRNA gene sequence similarity, respectively; Table 3) at exponential and late stationary algal growth phases reflects their high metabolic plasticity, considering the deep differences in terms of inorganic nutrient concentrations and organic matter quality and availability between the two distinct phases. *Oceanicaulis* representatives have been retrieved from several marine algal cultures (*Alexandrium tamarense*, Strompl, 2003; *Emiliania huxleyi*, Zabeti, Bonin, Volkman, Guasco, & Rontani, 2010; *Eutreptiella* sp., Kuo & Lin, 2013; *Ostreococcus tauri*, Abby, Touchon, De Jode, Grimsley, & Piganeau, 2014), and genes and regulons involved in biosynthesis pathways of B vitamins (i.e., B_1_, B_7_, and B_12_) have been detected in *Oceanicaulis* phylotypes (Oh et al., 2011). Additionally, the versatile chemoheterotrophic metabolism reported for this genus (Chen, Sheu, Chen, Wang, & Chen, 2012; Oh et al., 2011; Strompl, 2003) also encompasses efficient phosphate uptake capacity in carbon‐limited medium and inorganic nutrient depleted conditions through high‐affinity phosphate transporters located in the prosthecae (McAdams, 2006; Oh et al., 2011). *Dinoroseobacter shibae* strains have been firstly retrieved in association with toxic cultured benthic and planktonic dinoflagellates (i.e., *Alexandrium ostenfeldii*,* Prorocentrum lima*,* Protoceratium reticulatum*) and other nontoxic marine microalgae (reviewed in Wagner‐Döbler et al., 2010). High contributions of this species (>22%, Figure 4) were observed here all along the *O*. cf. *ovata* growth. A mutualistic‐pathogenic bimodal behavior in response to algal physiological status has been demonstrated for *D*. *shibae* in co‐culture with toxic dinoflagellates. Specifically, the bacterium is able to switch from a mutualistic phase, when it synthesizes vitamins B_1_ and B_12_ (Biebl et al., 2005) and antibacterial compounds primarily in exchange for the algal‐released dimethylsulfoniopropionate, to a pathogenic phase triggered by algal senescence signaling molecules (Wagner‐Döbler et al., 2010; Wang et al., 2014, 2015). A similar behavior has been found for other related Rhodobacteraceae (i.e., *Phaeobacter gallaeciensis*,* P. inhibens*, Seyedsayamdost et al., 2011; Segev et al., 2016; Wang et al., 2016; *Rugeria pomeroyi*, Riclea et al., 2012; *Silicibacter* sp., Sule & Belas, 2013). *O*. cf. *ovata* produces dimethylsulfoniopropionate (Vanucci, Pezzolesi, et al., 2016), whereas its vitamin requirements are still unknown. A focus on *O*. cf. *ovata* B vitamins demand and on the dinoflagellate's potential vitamin uptake through its associated bacterial community warrants future research, considering that many harmful dinoflagellates show auxotrophy for some B vitamins (Croft, Lawrence, Raux‐Deery, Warren, & Smith, 2005; Cruz‐López & Maske, 2016; Koch et al., 2014; Tang, Koch, & Gobler, 2010). In this study, *Roseovarius* accounted for almost 20% of the total bacteria at *O*. cf. *ovata* exponential growth phase. *Roseovarius*‐affiliated phylotypes have been recovered from different cultured marine algal species (Biebl et al., 2005; Onda et al., 2015), also concurrently with *Oceanicaulis* (Abby et al., 2014; Kuo & Lin, 2013) and *Fabibacter* relatives (Green, Echavarri‐Bravo, Brennan, & Hart, 2015), and in association with toxic dinoflagellate blooms (Vanucci, Guidi, et al., 2016; Yang et al., 2015). Metagenomic and biochemical analyses highlighted the large metabolic portfolio of *Roseovarius* (Bruns et al., 2013; Riedel et al., 2015), including synthesis of dual nature compounds (i.e., algal growth promoting and algicidal ones; Ziesche et al., 2015). However, *Roseovarius* as well as *Labrenzia* strains have been shown to require both vitamin B_1_ and B_7_ for the growth (Biebl, Lu, Schulz, Allgaier, & Wagner‐Döbler, 2007; Biebl et al., 2005).

The comparison between laboratory and environmental data reveals that the alphaproteobacterial consortium retrieved in *O*. cf. *ovata* cultures was phylogenetically closely related to that one found during the *O*. cf. *ovata* bloom, the latter composed by the Rhodobacteraceae *Ruegeria, Jannaschia,* and *Roseovarius* together with *Erythrobacter* (Vanucci, Guidi, et al., 2016). Besides, the members forming the two consortia altogether share comparable metabolic traits, including species‐specific de novo B vitamins synthesis and a bimodal behavior with the ability to synthesize both antibacterial and algicidal compounds (Newton et al., 2010; Pujalte, Lucena, Ruvira, Arahal, & Macián, 2014; Ziesche et al., 2015), suggesting some degree of functional similarity and redundancy. In fact, *Jannaschia* and *Ruegeria* phylotypes were still present in cultures, although in lower abundances (Table S3). Thus, while culture conditions partially modify the relative importance of lower‐order taxa composing the environmental bacterial community, the overall metabolic and functional profile seems someway maintained. Consistently with the field observation (Vanucci, Guidi, et al., 2016), additional metabolic abilities typical of the Alphaproteobacteria forming the two consortia further favored these phylotypes over other taxa also in cultures. In fact, *Dinoroseobacter* as well as *Roseovarius* and *Labrenzia* are aerobic anoxygenic photosynthetic bacteria (Biebl et al., 2005, 2007), known to outcompete strictly chemoheterotrophs when growing in a light‐dark carbon‐limited regime, becoming the most metabolically active bacteria (Koblížek, 2015; Soora et al., 2015; Wang et al., 2014, 2015). Moreover, *Dinoroseobacter* as well as *Oceanicaulis* and *Roseovarius* members are able to grow under anaerobic conditions (Laass et al., 2014; Oh et al., 2011; Riedel et al., 2015; Wagner‐Döbler et al., 2010), which likely occurred in *O*. cf. *ovata* mucilaginous aggregates at the stationary phase of the algal growth, as suggested by detection of ammonia in the culture medium (~3.0 μmol L^−1^ at both days 24 and 42; data not shown). Analogous diel anoxia conditions occur in natural *O*. cf. *ovata* mats (Vanucci, Guidi, et al., 2016). Conversely, a minor contribution of Gammaproteobacteria was found at all *O*. cf. *ovata* growth phases (<1%). Limited abundances of this class have been reported also during the *O*. cf. *ovata* natural bloom (<6%) and in association with other toxic dinoflagellates (e.g., *Alexandrium* spp., Jasti et al., 2005; Garcés et al., 2007; *Lingulodinium polyedrum*, Cruz‐López & Maske, 2016).

Within Bacteroidetes, bacterial diversity in cultures was dominated by Sphingobacteria (Figure 3), whereas Flavobacteria prevailed in the *O*. cf. *ovata* natural bloom (Vanucci, Guidi, et al., 2016). Although being considered metabolically and functionally similar (Kirchman, 2002; Teske, Durbin, Ziervogel, Cox, & Arnosti, 2011), in relation to phytoplankton Sphingobacteria have been found mainly associated with coccolithophores (Green et al., 2015; Van Oostende et al., 2008), whereas Flavobacteria with diatoms (Grossart et al., 2005; Teeling et al., 2012; Xing et al., 2015). Thus, Flavobacteria could have been more efficient in degrading phytodetritus from the epiphytic diatoms co‐occurring in relevant proportion during the *O*. cf. *ovata* bloom (Accoroni et al., 2016; Vanucci, Guidi, et al., 2016). According to secondary colonizer traits typical of Flammeovirgaceae (Kim et al., 2013; Nedashkovskaya & Ludwig, 2011), the high contribution of *Fabibacter* at *O*. cf. *ovata* mid stationary phase indicates the occurrence of high molecular weight compounds less susceptible to Rhodobacteraceae attack such as phytodetritus (Buchan et al., 2014) and mucus (de Castro et al., 2010). Recalcitrant carbon‐rich macromolecules like *O*. cf. *ovata* toxins (Pinna et al., 2015), are also known to be increasingly released from the exponential to the stationary phase (Pezzolesi et al., 2014, 2016; Vanucci, Pezzolesi, et al., 2012; Vanucci, Guerrini, et al., 2012). Successively, Rhodobacteraceae genera such as *Labrenzia* and *Dinoroseobacter* may have also responded to a renewed availability of low molecular weight compounds by Bacteroidetes algal‐derived matter remineralization (Buchan et al., 2014; Fernández‐Gómez et al., 2013; Teeling et al., 2012) at the algal stationary phase. Moreover, *Labrenzia alexandrii* (OTU #4, 100% similarity; Biebl et al., 2007) warrants further investigation since killing‐host activity also by R‐bodies has been hypothesized for this species (Fiebig et al., 2013).

Inhibitory/algicidal activity has been also strongly suggested for *Dietzia‐*affiliated members (Kim, Jeong, & Lee, 2008; Le Chevanton et al., 2013), which became abundant at the *O*. cf. *ovata* late stationary growth phase (OTU #16), consistently with the pattern generally observed for recalcitrant substrate degrading Actinobacteria in both algal cultures and natural outbreaks bacterial succession (Bagatini et al., 2014; Basu, Deobagkar, Matondkar, & Furtado, 2013; Vanucci, Guidi, et al., 2016). During the *O*. cf. *ovata* bloom, however, *Ilumatobacter* phylotypes were the main representatives of Actinobacteria at maintenance/decline phase (Vanucci, Guidi, et al., 2016), as found in diatoms degradation processes (Bagatini et al., 2014; Zakharova et al., 2013). This finding suggests a more intimate relationship between *Ilumatobacter* and diatoms co‐occurring at the bloom rather than with *O*. cf. *ovata*. Whereas, consistently with the results reported here, *Dietzia* relatives have been isolated during the termination of the planktonic harmful dinoflagellate *Cochlodinium polykrikoides* blooms (Kim et al., 2008), suggesting a more recurrent interaction of this bacteria with the dinoflagellates.

## 5 CONCLUSIONS

In this study, an alphaproteobacterial consortium composed by the Rhodobacteraceae *Dinoroseobacter* and *Roseovarius*, together with *Oceanicaulis*, was strongly associated with *O*. cf. *ovata* over the algal growth cycle. *Fabibacter* together with *Labrenzia* and *Dietzia* were relevant at late phases of the algal growth.

Overall, the bacterial successional pattern, and the metabolic and functional traits of the bacterial community selected under laboratory conditions mirror those ones underpinning *O*. cf. *ovata* bloom dynamics in field. In particular, bacterial community metabolic and functional profile appears primarily relying on the presence of genera encompassing mutualistic‐pathogenic bimodal behavior phylotypes, and on synergistic bacterial‐bacterial interspecific interactions for maximizing *O*. cf. *ovata* organic matter exploitation and fulfillment of the nutritional needs within the community. Thus, laboratory cultures appear a tractable system for unveiling environmental and anthropogenic factors which, besides affecting *O*. cf. *ovata* directly, could also induce shifts on *O*. cf. *ovata* bacterial community structure and dynamics, and connected changes in algal–bacterial interactions with subsequent cascading effects on bloom development and algal toxins production (Buchan et al., 2014).

In order to gain insight into the functional significance and metabolic exchanges underpinning these complex interactions, future experimentation is required in defined co‐cultures based on *O*. cf. *ovata* and bacterial isolates selected among those composing the algal associated community retrieved in this study. A focus on the bacterial phylotypes with an assessed mutualistic‐pathogenic bimodal behavior, in response to algal physiological status, which could have relevance in *O*. cf. *ovata* bloom initiation and termination phases, it is suggested.

With respect to viral lytic activity, bacterial abundance pattern and bacterial successional trend found in this study suggest investigation on viral host specificity for the most abundant Alphaproteobacteria associated with *O*. cf. *ovata*, particularly at the first bacterial growth step. An exception can be made with regard to *D. shibae*, known to harbor the most complex Rhodobacteraceae’ viral defense system retrieved to date (Wagner‐Döbler et al., 2010). At the same time, the presence of viruses specific for *O*. cf. *ovata* and their forms of infection (Sime‐Ngando, 2014) should be also investigated.

## CONFLICT OF INTEREST

The authors declare that they have no conflict of interest.

## ETHICAL APPROVAL

This article does not contain any studies with human participants or animals performed by any of the authors.
