# Supplementary material for: Microbial dynamics during harmful dinoflagellate Ostreopsis cf. ovata growth: Bacterial succession and viral abundance pattern
Source: Microbiologyopen. 2018 Feb 27;7(4):e00584. doi: 10.1002/mbo3.584 (PMC6079179; doi:10.1002/mbo3.584)

Supplementary Material

Microbial dynamics during harmful dinoflagellate *Ostreopsis* cf. *ovata* growth: bacterial succession and viral abundance pattern

Flavio Guidi, Laura Pezzolesi, Silvana Vanucci*

* Corresponding author, Department of Chemical, Biological, Pharmaceutical and Environmental Sciences (ChiBioFarAm), University of Messina, Viale Ferdinando Stagno d'Alcontres 31, 98166 S. Agata, Messina, Italy. Tel.: +39 347 5714315; fax: +39 (0)544 937411
E-mail address: silvana.vanucci@unime.it

Table S1. Relative abundance (%) of all bacterial phyla detected by ION torrent sequencing analysis in *O.* cf. *ovata* batch cultures at the time of inoculum (day 0) and during the different algal growth phases (days 6, 24 and 42). Rare phyla accounted for less than 1%.

Phylum	% in at least one sample	day 0	day 6	day 24	day 42
Proteobacteria	≥ 1	87.81	97.70	66.13	87.57
Bacteroidetes	≥ 1	12.04	2.21	33.11	9.94
Actinobacteria	≥ 1	0.04	0.04	0.71	2.46
Spirochaetes	rare	0.04	0.01	0.04	0.03
TM7	rare	0.07	0.04	0.01	0.00

Table S2. Relative abundance (%) of all bacterial classes detected by ION torrent sequencing analysis in *O.* cf. *ovata* batch cultures at the time of inoculum (day 0) and during the different algal growth phases (days 6, 24 and 42). Rare classes accounted for less than 1%.

Class	% in at least one sample	day 0	day 6	day 24	day 42
Alphaproteobacteria	≥ 1	86.93	96.88	65.73	87.00
Sphingobacteria	≥ 1	11.97	2.16	32.98	9.78
Actinobacteria	≥ 1	0.04	0.04	0.71	2.46
Betaproteobacteria	rare	0.16	0.11	0.22	0.28
Flavobacteria	rare	0.08	0.05	0.14	0.17
Gammaproteobacteria	rare	0.78	0.74	0.18	0.28
Spirochaetes	rare	0.04	0.01	0.04	0.03

Table S3. Relative abundance (%) of all bacterial genera detected by ION torrent sequencing analysis in *O.* cf. *ovata* batch cultures at the time of inoculum (day 0) and during the different algal growth phases (days 6, 24 and 42). Rare genera accounted for less than 1%.

Genera	% in at least one sample	day 0	day 6	day 24	day 42
Oceanicaulis	≥ 1	40.25	34.96	14.16	32.94
Dinoroseobacter	≥ 1	22.23	35.43	25.19	27.70
Labrenzia	≥ 1	5.56	6.85	24.29	23.04
Roseovarius	≥ 1	17.76	18.34	7.41	5.68
Sphingopyxis	≥ 1	2.09	1.38	0.66	0.97
Fabibacter	≥ 1	7.38	0.67	25.22	5.21
Balneola	≥ 1	2.96	1.00	1.06	0.25
Dietzia	≥ 1	0.04	0.04	0.82	1.67
Alcanivorax	rare	0.39	0.62	0.04	0.05
Corynebacterium	rare	0.00	0.00	0.00	0.98
Donghicola	rare	0.00	0.01	0.10	0.05
Flavisolibacter	rare	0.06	0.08	0.00	0.01
Glaciecola	rare	0.06	0.05	0.08	0.09
Jannaschia	rare	0.00	0.01	0.00	0.00
Limnobacter	rare	0.17	0.12	0.25	0.30
Polaribacter	rare	0.04	0.01	0.07	0.05
Pseudomonas	rare	0.36	0.11	0.01	0.13
Reichenbachiella	rare	0.21	0.14	0.57	0.67
Rhizobium	rare	0.37	0.13	0.02	0.05
Ruegeria	rare	0.01	0.03	0.02	0.11
Spirochaeta	rare	0.05	0.01	0.05	0.03

Figure S1. Nitrate (NO_3^-) and phosphate (PO_4^{3-}) concentrations measured in *O.* cf. *ovata* culture medium. Each point is the mean of triplicate cultures. Bars indicate standard deviations.

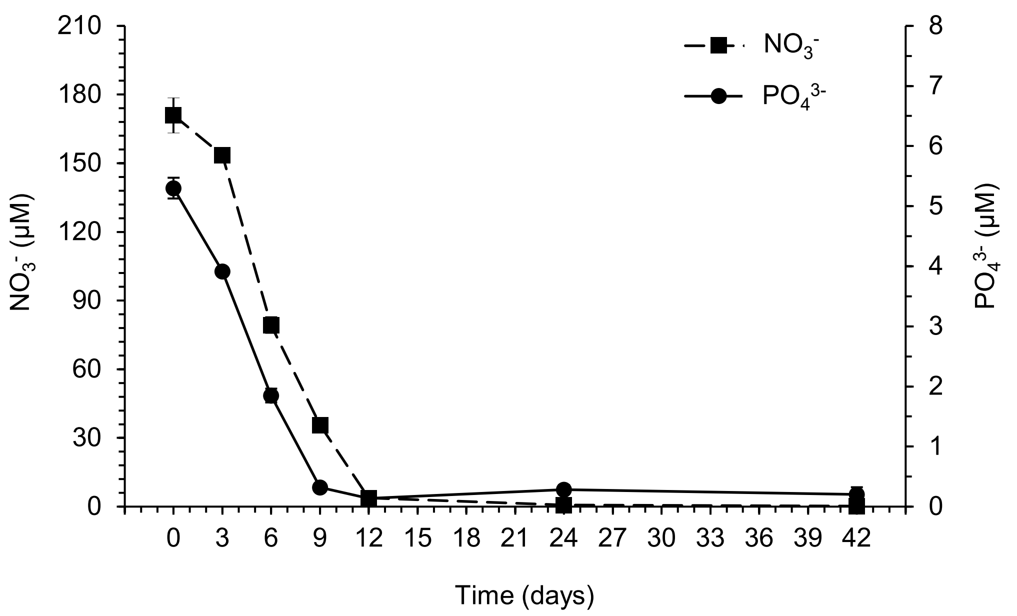

Figure S2. Virus to bacteria ratio (VBR) during the *O*. cf. *ovata* growth. Each value is the mean of triplicate cultures. Bars indicate standard deviations.

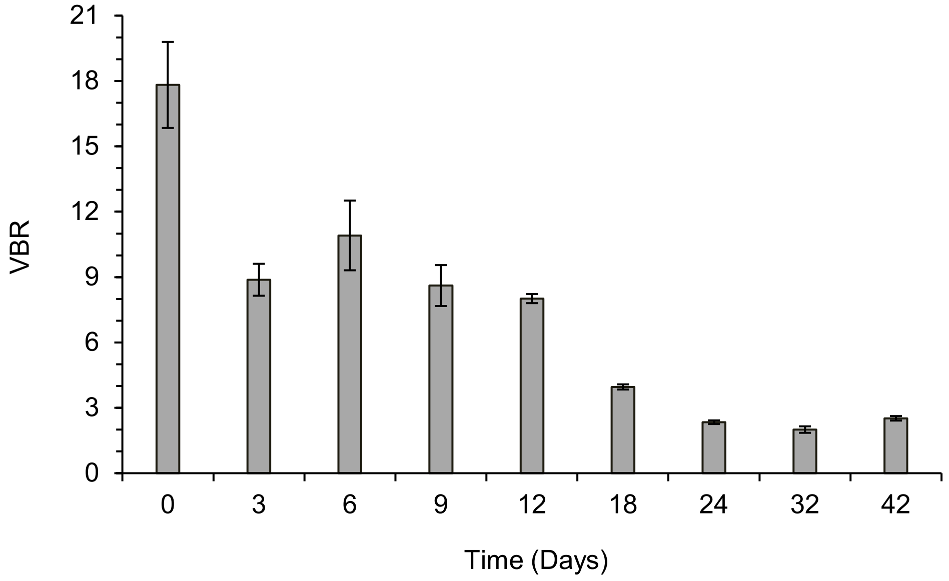

Figure S3. Diversity of the bacterial community associated with *O.* cf. *ovata* in batch cultures at the time of inoculum (day 0) and during the different algal growth phases (days 6, 24 and 42) as revealed from ION torrent sequencing data. Rarefaction curves were computed on bacterial OTUs at a dissimilarity level of 3%.

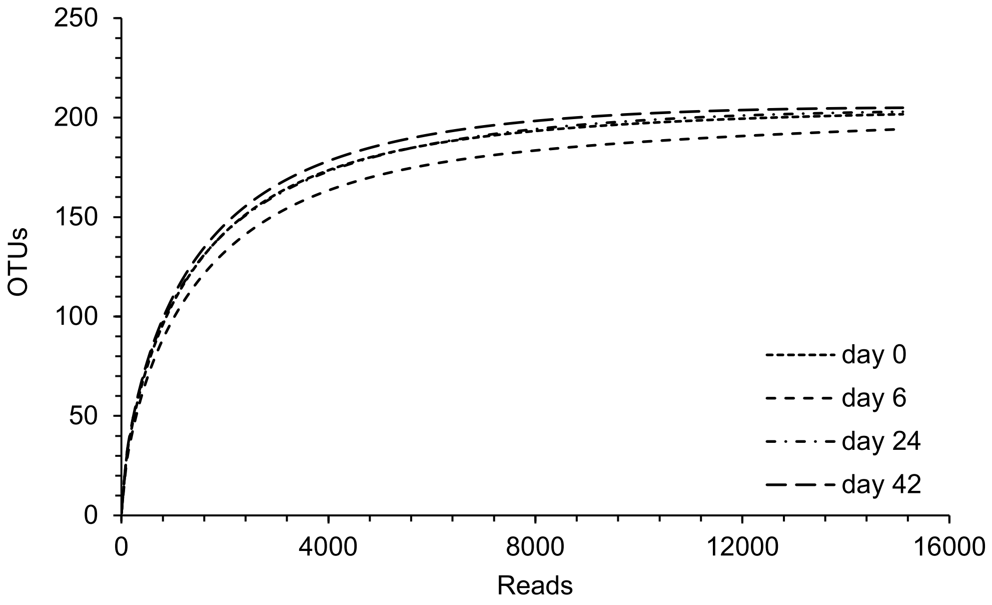

Supplement: Supplementary file 1 [file MBO3-7-e00584-s001.pdf]
